# Dietary Stimuli, Intestinal Bacteria and Peptide Hormones Regulate Female *Drosophila* Defecation Rate

**DOI:** 10.3390/metabo13020264

**Published:** 2023-02-12

**Authors:** Katerina Kotronarou, Anna Charalambous, Amalia Evangelou, Olympiada Georgiou, Andri Demetriou, Yiorgos Apidianakis

**Affiliations:** Department of Biological Sciences, University of Cyprus, P.O. Box 20537, Nicosia 2109, Cyprus

**Keywords:** fecal, starvation, enteroendocrine cells, visceral muscle, *Drosophila* genetic reference panel, Allatostatin, neuropeptide

## Abstract

Peptide hormones control *Drosophila* gut motility, but the intestinal stimuli and the gene networks coordinating this trait remain poorly defined. Here, we customized an assay to quantify female *Drosophila* defecation rate as a proxy of intestinal motility. We found that bacterial infection with the human opportunistic bacterial pathogen *Pseudomonas aeruginosa* (strain PA14) increases defecation rate in wild-type female flies, and we identified specific bacteria of the fly microbiota able to increase defecation rate. In contrast, dietary stress, imposed by either water-only feeding or high ethanol consumption, decreased defecation rate and the expression of enteroendocrine-produced hormones in the fly midgut, such as Diuretic hormone 31 (Dh31). The decrease in defecation due to dietary stress was proportional to the impact of each stressor on fly survival. Furthermore, we exploited the Drosophila Genetic Reference Panel wild type strain collection and identified strains displaying high and low defecation rates. We calculated the narrow-sense heritability of defecation rate to be 91%, indicating that the genetic variance observed using our assay is mostly additive and polygenic in nature. Accordingly, we performed a genome-wide association (GWA) analysis revealing 17 candidate genes linked to defecation rate. Downregulation of four of them (*Pmp70*, *CG11307*, *meso18E* and *mub*) in either the midgut enteroendocrine cells or in neurons reduced defecation rate and altered the midgut expression of *Dh31*, that in turn regulates defecation rate via signaling to the visceral muscle. Hence, microbial and dietary stimuli, and *Dh31*-controlling genes, regulate defecation rate involving signaling within and among neuronal, enteroendocrine, and visceral muscle cells.

## 1. Introduction

Normal gastrointestinal (GI) motility is a vital process in all living organisms, primarily implicated in food digestion and nutrient absorption, thus necessary for energy homeostasis [1,2]. It is also critical for host-microbiota interaction and host defense to infection [3,4]. Gastrointestinal motility is physiologically regulated by smooth muscle contractility, that is in turn controlled by extrinsic parasympathetic and sympathetic neurons, intrinsic enteric sensory and motor neurons, and GI hormones [5,6,7]. These are influenced by extrinsic factors, such as diet, medications, and infections, as well as intrinsic factors, such as gut–brain communication, inflammatory and degenerative processes, and overstimulation of visceral sensory pathways altering smooth muscle contractility and enteric nervous system function [8]. However, the molecular mechanisms involved, remain incompletely understood.

*Drosophila melanogaster* is a suitable model organism to study the regulation of intestinal physiology via the gut–brain communication axis by revealing basic concepts potentially applicable to various aspects of human intestinal pathology [9,10]. Despite the physiological divergence between vertebrates and insects, modeling of human intestinal diseases is possible in *Drosophila* due to its high degree of conservation with mammals in terms of signaling pathways controlling intestinal development, regeneration, and disease [9]. Furthermore, various human intestinal pathogens and alterations in intestinal microbiota can infect flies and cause intestinal pathology [11]. More importantly, the availability of genome-wide RNAi libraries, gene markers, and gene manipulation tools, render *Drosophila* easily amenable to reverse genetics.

*Drosophila* assays simultaneously provide the complex cellular composition of a model intestine along with the opportunity to assess organismal physiology faster and cheaper [9,12]. For example, insights have been provided using *Drosophila* to reveal mechanisms underlying gut motility, feeding behavior and energy mobilization involving the gut-brain communication axis [10]. Larval gut motility (peristalsis) is determined by the action of enteroendocrine cells (EEs) located anterior to the acidic stomach region [13]. Similarly, adult fly midgut muscle contraction is regulated by midgut EEs [14]. Enteroendocrine cells secrete various regulatory peptides, including Diuretic Hormone 31 (DH31) [15], the fly homolog of the vertebrate Calcitonin Gene-Related Peptide (CGRP), that is necessary and sufficient for larval gut peristalsis and adult midgut visceral muscle (VM) contractions [13,16]. The DH31 promotes VM contractions within 2 h of ingestion of pathogenic bacteria facilitating gut clearance of the ingested bacteria [14].

Furthermore, midgut EEs and neurons regulate feeding behavior and energy mobilization through glucagon-like adipokinetic hormone (AKH) and *Drosophila* insulin-like peptides (dILPs). Neuropeptide F (NPF; orthologous to mammalian neuropeptide Y) and its receptor influence total food intake indirectly by regulating food choice behavior [17,18] and lipid-metabolism [19,20]. Neurons producing Allatostatin A (AstA) participate in a circuitry that negatively regulates feeding behavior via metabolic changes controlling satiety [21,22]. Allatostatin A and its receptor DAR2 (AstA-R2), are regulated differentially by dietary carbohydrates and proteins, and AstA-neuronal activity modulates feeding choices between carbohydrates and proteins by regulating the balance between dILPs and AKH [23]. Moreover, midgut EEs sense nutrient stress through TOR signaling and secrete Allatostatin C (AstC), a *Drosophila* peptide hormone, homologous to mammalian somatostatin. Allatostatin C induces secretion of AKH to coordinate food intake and energy mobilization [24,25]. Tachykinin (Tk) and Myosuppressin (Ms) have modulatory roles on motility; the former confers both excitatory and inhibitory effects on gut motility [26], whereas the latter relaxes crop muscles, to allow expansion and increased food intake [10]. However, external stimuli and gene networks coordinating gut motility via the neuron-gut axis remain poorly defined.

The objectives of this study were to (a) determine the effect of dietary and microbial stimuli on the defecation rate of female *Drosophila* using a customized assay that served as a proxy of intestinal motility, (b) determine the heritability of this trait when using our assay and (c) identify novel genes through a genome wide association (GWA) analysis that interact with known peptide hormones, such as Diuretic hormone 31, forming gene networks implicated in the regulation of intestinal motility.

## 2. Materials and Methods

### 2.1. Preparation of LB and BHI Agar Plates with or without Rifampicin

Luria–Bertani (LB) agar (Invitrogen—Waltham, MA, USA) plates were prepared according to manufacturer’s instructions. A total of 32 g LB agar powder/1 L ddH_2_O was autoclaved and used in plain petri dishes. The LB agar rifampicin plates were prepared with the addition of 100 μg/mL rifampicin (Sigma–Aldrich, St. Louis, MO, USA, R3501) after autoclaving.

The BHI (Brain Heart Infusion) agar plates were prepared according to manufacturer’s instructions. A total of 37 g BHI (HIMEDIA, Einhausen, Germany) plus 15 g agar/1 L ddH_2_O was autoclaved and supplemented with 5 mg hemin (Sigma–Aldrich, St. Louis, MO, USA), 0.1 mg vitamin K1 (Sigma–Aldrich, St. Louis, MO, USA), and preferentially 100 μg/mL rifampicin (Sigma–Aldrich, St. Louis, MO, USA) after autoclaving. All plates were kept at 4 °C until usage.

### 2.2. Bacteria Strains, Infection and Determination of Bacterial Load

#### 2.2.1. Bacteria Strains

Gram-negative bacteria *Pseudomonas aeruginosa* (PA14) have been described previously [27]. Gram-positive bacteria *Enterococcus haemoperoxidus* and *Staphylococcus arlettae* are *Drosophila* isolates from OR flies (this study). These were maintained as glycerol stocks and stored at −80 °C.

#### 2.2.2. Bacterial Infection

A sample of 3 mL of the overnight cultures of either *P. aeruginosa* (PA14), or *E. haemoperoxidus* or *S. arlettae* alone, or in various combinations, were diluted 1:100 in LB (Lysogeny Broth) to prepare the overday 3 mL cultures. When the cultures reached OD_600_ = 3, they were used to prepare the infection mixtures (5 mL per vial: 3.5 mL ddH_2_O, 1 mL 20% sucrose and 0.5 mL bacteria OD_600_ = 3) or the control sucrose mix (5 mL per vial control: 4 mL ddH_2_O and 1 mL 20% sucrose). A total of 5 mL of the infection or control mix was used to soak a cotton ball in a narrow fly vial, which was subsequently plugged with a dry cotton ball. After 4–5 h starvation in empty vials, the flies were put in the infection vials and incubated at 25° with the cotton plug facing down.

#### 2.2.3. Bacterial Load

The number of bacteria colony forming units (CFUs) in five populations of OR flies, reared in parallel for a year, were determined either at 2 days following PA14 ingestion, or without as a control, at 25 °C. Flies were externally sterilized by brief dipping into pure ethanol, dried and placed into 2 mL Eppendorf tubes containing 200 μL lysogeny broth (LB) and a stainless-steel bead of 5 mm diameter (Qiagen, Hilden, Germany). Flies were homogenized using the TissueLyser II (Qiagen, Hilden, Germany) at 50 Hz for 5 min. Then LB or BHI was then added into the tubes containing the tissue lysate to reach the volume of 1000 μL. Serial dilutions of the lysate obtained from three flies were plated onto LB or BHI agar plates with or without 100 μg/mL rifampicin (Sigma-Aldrich, St. Louis, MO, USA) and incubated overnight at 37 °C. In total, bacterial colonies from three replicates per OR population were counted.

#### 2.2.4. Isolation of Bacterial Species from *Drosophila*

To analyze different bacterial species from the gut microbiota of *Drosophila*, two populations of Oregon-R female flies (OR3 and OR9) were surface sterilized by washing with 10% bleach, 70% ethanol and PBS before homogenization and plating on LB and BHI agar plates with 100 μg/mL rifampicin or not as a control. The plates were incubated at 28 °C for 4 days and single colonies were picked and isolated on new agar plates for three rounds to obtain pure cultures. These were then stored in glycerol stocks for microbiological experiments and colony PCR.

#### 2.2.5. Single Colony PCR and Analysis of 16S rRNA Genes

Of the different pure cultures, single colonies were picked and transferred into PBS buffer containing 200 μg/mL Proteinase K and 10 mg/mL Lysozyme and incubated for 30 min at 37 °C and 2 min at 95 °C. The samples were centrifuged for 2 min at 13,000 rpm and the supernatant transferred to a new vial. The 16S rRNA Gen was amplified using the GM3F and GM4R primers [28], using the Phusion Polymerase (New England Biolabs, Ipswich, MA, USA) which produced a product of about 1500 bp. These PCR products were then ligated into the TOP TA Vector (TOPO TA Cloning Kit for Sequencing, Invitrogen, Waltham, MA, USA) and transformed into chemocompetent *E. coli* DH5alpha according to the manufacturer’s instructions. The vector including the insert was extracted from *E. coli* and the DNA sequence was subjected to BLAST analysis to identify the isolated bacterial species.

#### 2.2.6. DNA Extraction from Bacterial Species for Sequencing

The DNA extraction was performed using the *QiaAmp DNA Mini* kit (Qiagen, Hilden, Germany) according to the manufacturer’s recommendation, with the following modifications. Briefly, an inoculation loop was used to pick bacterial colonies from the pure cultures grown on LB or BHI +/− rifampicin agar plates and the bacteria were resuspended in gram-positive lysis buffer (20 mg/mL lysozyme; 20 mM Tris·HCl, pH = 8.0; 2 mM EDTA; 1.2% Triton^®^). The following lysis and purification steps were performed according to the kit’s protocol for DNA extraction from gram-positive bacteria.

### 2.3. Drosophila Melanogaster Diet, Maintenance, Strains and Experiments

#### 2.3.1. *Drosophila* Diet and Maintenance 

All strains and crosses were reared on a standard agar/cornmeal diet (1% Agar, 3% Yeast, 5% Sugar, 6% Cornmeal, supplemented with 2.56% Tegosept and 0.38% Propionic Acid) and kept in plastic bottles, with approximately 50 mL of fresh fly food in a 12-h light-dark cycle in a temperature-controlled incubator (Fitotron from Weisstechnik, Reiskirchen-Lindenstruth, Germany) at 25° (unless specified otherwise) with 65% humidity. For maintenance flies were transferred to new bottles with fresh fly food every 3 to 4 days. For fly infection experiments flies were transferred to clean vials with fresh fly food every day, containing preservatives (propionic acid and Tegosept), to help eliminate microbiota and avoid pre-treatment with antibiotics that would interfere with subsequent bacterial infection and colonization. For the Drosophila Genetic Reference Panel (DGRP) screening for fecal spot number, prior to infection, female mated flies were aged for 4 days at 25 °C in bottles changed daily containing fly food supplemented with 50 ug/mL of the broad-range antibiotic Rifampicin, which does not kill PA14, but can eliminate most of the microorganisms present in the intestine of the flies.

#### 2.3.2. Germ-Free Flies

Female flies were transferred in empty bottles covered with a fruit juice agar plate (35 × 10 mm). The fruit juice agar plate was prepared following boiling of 2% agar dissolved in fruit juice and supplemented with Tegasept and propionic acid to final concentrations of 0.56% and 0.37%, respectively. Once the mixture was solidified, 0.2 mL of yeast paste (66% dry yeast dissolved in double-distilled H_2_O) was transferred into the middle of each Petri dish. Flies were conditioned by feeding on fruit juice agar plates for a day before being transferred into clean bottles with freshly prepared fruit juice agar plates on the top. After a 15 h incubation at 25 °C, the eggs were collected into a mesh basket using a brush. Each basket was placed in a beaker containing 20 mL of 50% bleach for a maximum of 2 min or until ~80% of dorsal appendages were dissolved as a result of removal of the chorion layer. Bleached eggs were then washed with sterile double-distilled H_2_O under the microbiological hood and transferred into bottles containing sterile fly food and maintained at 25 °C. Once the offspring began to emerge, they were transferred into bottles with sterile food. Lysates obtained from the emerged flies were plated onto LB media and incubated at 37 °C overnight to ensure that they were germ-free.

#### 2.3.3. *Drosophila* Strains

Oregon-R flies were used as a standard wild-type strain in experiments assessing effects of infection, gut microbiota, and metabolic stress (ethanol in the diet and starvation), on the defecation rate. In some experiments, explicitly indicated within this work, OR flies reared in parallel for one year were sampled and assessed. The DGRP collection of wild type inbred sequenced strains [29,30] was used in the screen to assess defecation rate as a measure of fecal spots. The VDRC *UAS-RNAi* lines used in this study to examine the effects of ubiquitous or site-specific (EE or pan-neuron or visceral muscle) downregulation of GWAS-identified and hormone-encoding genes on the defecation rate and gene expression in the midgut and head were the following: 110698/KK targeting *Pmp70*, 30050/GD and 108230/KK targeting *CG11307*, 17444/GD targeting *meso18E*, 110581/KK targeting *mub*, 104119/KK targeting *CG32365*, 7789/GD targeting *dom*, 18177/KK targeting *NK7.1*, rad targeting *CG42629*, 1402/GD and 1403/GD targeting *hh*, 107945/KK and 27117/GD targeting *CG7166*, 100915/KK targeting *CG8065*, 12610/GD targeting *jumu*, 101090/KK targeting *Bin3*, 25222/GD targeting *ckn*, 50295/GD targeting *Dh31*, 1439816/GD and 103215/KK targeting *AstA*, 102735/KK and 13773/GD targeting *AstC*, 108760/KK targeting *Ms*, 330662/KK and 108772/KK targeting *NPF*, 103662/KK targeting *Tk*, 106076/KK and 5294/GD targeting *AstB*, 108648/KK and 1327/GD targeting *DAR2*, 9199/GD and 106512/KK targeting *ilp3*. The Gal4 lines were crossed to *w^1118^*, as a control. Other stocks used in this study were the following (source and/or stock center numbers in parentheses): *UAS-Dh31^RNAi^* (BDSC#41957), *UAS-Dh31-R^RNAi^* (BDSC#25925), *w;actin-Gal4*, *UASGFP/CyO* (actin-Gal4), *w;pros^V1^-Gal4/TM6C* (Prospero-Gal4), *w;elav-Gal4/FM7i UAS-dsRed/Cyo* (elav-Gal4) and *24B-Gal4 UAS-GFP/TM3*. The *Dh31-Gal4* (KII) and *w;UAS-mCherryNLS* flies were gifts from Armel Gallet.

#### 2.3.4. Defecation Assay

Female flies were starved in empty vials for 5 hr and then placed in vials with cotton balls impregnated with 5 mL 4% sucrose and 0.5% *w*/*v* bromophenol blue (BPB; Scharlab, Barcelona, Spain)) adjusted to pH = 7. Fifty flies were split into three vials with BPB and were allowed to feed for 5 h (vials were placed in the incubator with the cotton plug facing down). Ten flies from each vial were moved to a petri dish containing a sterile cotton ball impregnated with 2.5 mL of the BPB solution and were left to defecate for 20 h. Then, the flies were removed from the plates and the total number of fecal spots was measured in the three plates. Fecal spots per fly per day are plotted. All experiments were performed at 25 °C.

For the assessment of defecation rate under infection conditions, female flies were fed concentrated bacteria of OD_600_ = 50 using an overday culture of OD_600_ = 2 concentrated 25 times by centrifugation and resuspension of the bacterial pellet in 4% sucrose. Specifically, each feeding vial plugged with a cotton ball contained 5 mL agar gel (3% *w*/*v* agar in H_2_O) on top of which a Whatmann disc with 200 μL of the infection mix was placed. Twenty-five mated starved young female flies per vial were allowed to feed for 20 h on either infection mix or 4% sucrose (as a control) at 25 °C, and their fecal spots were then counted and plotted per fly per day.

For assessment of the defecation rate under starvation conditions, the control group was given 0.5% BPB in 4% sucrose, as above, whereas the test-group (under starvation stress) was given 0.5% BPB in water (final pH of both BPB solutions adjusted to pH = 7). Ten control (no starvation stress) flies were transferred directly from fly food in a vial for 5 h to polystyrene vials, each containing a sterilized cotton ball pressurized at the bottom of the vial and soaked with 5 mL BPB in 4% sucrose, while 10 test (water-only) flies were transferred in a polystyrene vial containing a cotton ball soaked with 5 ml BPB in water. Then each group was transferred to a polystyrene petri dish (10 cm in diameter) containing a half (scissors cut) sterilized cotton ball soaked with 2.5 mL of the corresponding BPB solution. Twenty-four hours later, flies were discarded from the petri dishes and the excreta of *n* = 10 flies per replicate per 24 h were assessed. Data were collected from 6 biological replicates and fecal spots per fly per 24 h are plotted.

#### 2.3.5. Survival Assay

Young female flies were subjected to metabolic stress conditions—either starvation or ethanol consumption. Sterile glass tubes were used and Whatman filter paper was placed at the bottom of each tube. The ethanol was administered on Whatman paper in solution with 4% sucrose. For starvation, the Whatman paper was only soaked with water. As a control, the flies were kept in vials with a 4% sucrose solution. The percentage of dead flies was calculated daily as the (number of dead flies per vial/total number of flies) × 100 until all flies were dead in each vial. The time when 50% of the flies were dead (LT50%, lethal time 50%), was used as an indicator of survival for comparisons.

#### 2.3.6. Determination of Narrow-Sense Heritability (*h^2^*)

Eight crossing schemes of parental and F1 and F2 generations were performed: four between combinations of DGRP strains displaying high defecation rates (25201, 28171 and 25208) and four between combinations of DGRP strains displaying low defecation rates (28182, 28150 and 28153). Both the parents’ and offspring’s defecation rates were determined. The variable *h^2^* was determined for each crossing scheme using the formula $h^{{}^{2}}=({\bar{x}}_{F1}-{\bar{x}}_{pop})÷({\bar{x}}_{par}-{\bar{x}}_{pop})$, defined as the mean defecation rate of the *F*1 generation of a given crossing scheme (${\bar{x}}_{F1}$) minus the population mean (${\bar{x}}_{pop}$), that is the average defecation rate of F2 generations of all 8 crossing schemes, divided by the mean defecation rate of the two parental strains of the given crossing scheme (${\bar{x}}_{par}$) minus the population mean (${\bar{x}}_{pop}$).

#### 2.3.7. Fly Midgut and Head Dissections

Fly midgut and head dissections were performed as previously described [27]. Briefly, flies were anesthetized (using CO_2_) and placed on ice to stay under anesthesia. Midguts and heads of flies were dissected in 1× PBS (130 mM NaCl, 30 mM NaH_2_PO_4_, 70 mM Na_2_HPO_4_) and immediately placed in dry ice and stored at −80 °C for later use.

### 2.4. RNA Extraction, cDNA Synthesis and RT-qPCR

The RNA was extracted from 20 midguts and 20 heads per strain per condition per biological replicate using Qiazol (Qiagen). More specifically, 500 μL Qiazol was used per 20 midguts, followed by repeated pipetting and 1 mL Qiazol was used per 20 heads, followed by addition of metal beads and use of the TissueLyser LT machine for 20 min. We used 800 ng of total RNA to synthesize the cDNA using the RQ1 RNase-Free DNase Kit (Promega) according to the manufacturer’s protocol. Reverse transcription was performed using total DNase-treated RNA and the TaKaRa Prime Script RT Master Mix Kit. The qPCR amplification was performed using gene-specific primers with the following amplification program: 95 °C for 2 m (initial denaturation), 40 cycles of 95 °C for 10 s (denaturation), 60 °C for 30 s (annealing, extension) and 65 °C for 1 min (final extension). Primer sequences for each gene are shown in Supplementary Table S1. Expression of the genes of interest was normalized to the expression levels of two reference genes, *rpl32* and *gapdh1*, using the 2^−ΔΔCt^ method. Data were analyzed using the Bio-Rad CFX Manager 3.1 program.

### 2.5. Immunohistochemistry

Dissected midguts were fixed with 4% formaldehyde (FA) for 30 min and rinsed three times with 1× PBS. Blocking was with 1× PBS, 0.2% Triton-X, 0.5% BSA for 20 min. Primary antibody was mouse anti-Prospero (1:100; MR1A, DSHB, RRID: AB-528440), incubated overnight in the dark at 4 °C. Midguts were washed three times for 10 min in 1× PBS containing 0.2% Triton-X. Secondary antibody against mouse conjugated to Alexa Fluor 488 (DaM488 1:1000, Alexa Fluor^™^ #A-21202. RRID AB-141607) were used at 1:1000. Samples were incubated in secondary antibody solution for 2 h at room temperature in the dark, with mild shaking. Midguts were washed three times, mounted on glass microscope slides in 20 μL of Vectashield (Vector), covered with glass coverslips and sealed with nail polish.

### 2.6. Image Acquisition and Analysis

Stacks of optical sections were acquired using the Leica TCS SP2 DMIRE2 confocal microscope. Prospero positive EE cells were counted under the fluorescent microscope (Zeiss Axioscope A.1) at 20× magnification along the whole midgut. For regional assessment of anterior and posterior midgut, a standard frame of per midgut region per was captured and cropped into 300 × 166 µm.

### 2.7. Statistical Analysis

The *Z*-value of each DGRP line (standard deviations above or below the mean) was calculated by subtracting the average number of fecal spots of all 153 strains from the fecal spots of the line and dividing by the standard deviation of the fecal spot number of all 153 strains. For statistical analysis of fly survival, the Kaplan–Meyer method was applied, using the log-rank test (MedCalc statistical software, Ostend, Belgium). For statistical analysis of the defecation rate, the Mann–Whitney U test was applied to groups of six values. When >10 values per sample were available the two-tailed Student’s *t*-test was used for pairwise comparisons. For statistical analysis of CFUs, we used two-way ANOVA test with Tukey’s post-hoc correction. The RT-qPCR experiments were performed in six biological replicates, two technical replicates each, analyzed via *t*-test. Error bars throughout represent standard deviation of the mean. Significance is indicated by * *p* < 0.05, ** *p* < 0.01, *** *p* < 0.001; ns, not statistically significant.

## 3. Results

### 3.1. Specific Gut Microbiota Composition and Virulent Bacteria Ingestion Increase Defecation Rate in Drosophila Females

**Figure 1:**
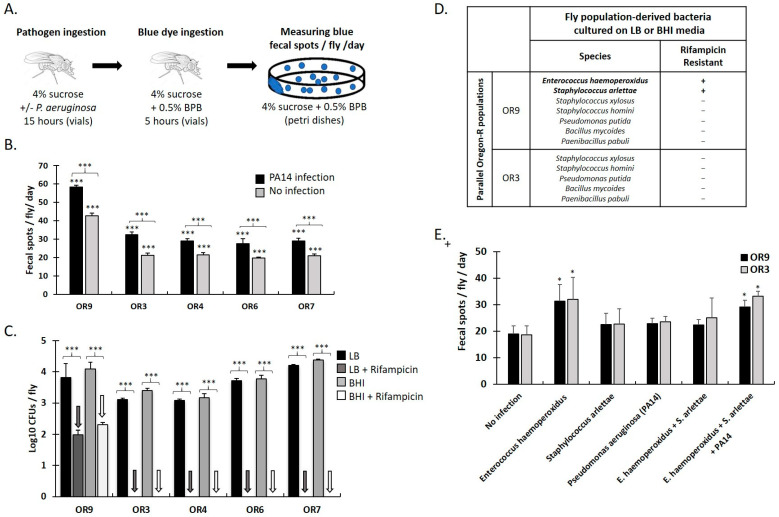
Ingestion of virulent bacteria and specific gut microbiota increase gut motility in *Drosophila*. (**A**) Defecation assay customized to examine the impact of PA14 on the defecation rate. At 15 h of feeding on PA14 or vehicle (4% sucrose) female flies were starved in empty fly vials for 5 h and then allowed to feed again for 5 h in fly vials containing 4% sucrose and 0.5% bromophenol blue (BPB) which colored fly food blue (at pH = 7). Conditioned flies were then transferred to plates feeding on 4% sucrose and 0.5% BPB for another day. Defecation rate was calculated as fecal spots per fly per day excreted by groups of 10 flies in 6 independent replicates. (**B**) Fecal spots per fly per day upon PA14 infection or without infection, of five populations of Oregon-R (OR) flies, reared in parallel in the lab for one year. Statistical analysis was performed using two-way ANOVA test with Tukey’s post-hoc correction. Statistical significance was observed in all cases (*** *p* < 0.001). (**C**) CFUs of intestinal bacteria per fly, shaped on LB or BHI media, in the absence or presence of rifampicin. Statistical analysis was performed using two-way ANOVA test with Tukey’s post-hoc correction. Statistical significance was observed in all cases (*** *p* < 0.001). (**D**) Bacterial species found in adult female flies of the OR9 and OR3 populations feeding on LB or BHI media, with and without rifampicin. Extraction of genomic DNA from single colonies and sequencing followed by BLAST search of the sequences, revealed five rifampicin-sensitive bacterial species, common to both OR9 and OR3 and two rifampicin-resistant strains (*E. haemoperoxidus* and *S. arlettae*), exclusive to OR9. (**E**) Fecal spots per day per female of the OR9 and OR3 populations, following ingestion of either PA14 or rifampicin-resistant strains *E. haemoperoxidus* or *S. arlettae*, alone or in combinations, in six independent replicates. Statistical analysis was performed using the Mann–Whitney U test. Statistical significance is indicated as * *p* < 0.05. All error bars represent standard deviation of the mean.

We customized a *Drosophila* gut motility proxy assay that enables fast, accurate and reproducible assessment of the deposition rate of female excreta (fecal spots per fly per day), as a function of dietary input, ingested or indigenous microbes, and host genetic background (Figure 1A).

We have previously established that *P. aeruginosa* and other bacteria found in humans can colonize and damage the fly intestine. Some of them can also kill *Drosophila* within a few days, while others within three weeks [11]. Populations of Oregon-R (OR) flies, reared in parallel in the lab for one year, were sampled and assessed while untreated or upon ingestion of an infection mix containing *P. aeruginosa* (strain PA14). The defecation rate of OR flies sampled from one population (hereunto referred to as OR9) was approximately two-fold higher, compared to that of all other sampled OR populations examined (Figure 1B). Moreover, for all sampled OR populations, the defecation rate was increased upon ingestion of PA14 by an average of 29% (Figure 1B). To examine whether the higher defecation rate in OR9 flies was due to differences in the number or the composition of intestinal bacteria, the CFUs developed on LB and BHI media were measured in the absence or presence of rifampicin, a broad-spectrum bactericidal antibiotic [31,32]. Figure 1C shows that flies from all OR populations harbored 3.1–4.2 and 3.2–4.4 log_10_ CFUs per fly, assessed using LB and BHI plates, respectively, without using an antibiotic. Rifampicin addition in plate media eliminated CFUs from all plates, except those of the OR9 population samples, which harbored 1.9 and 2.3 log_10_ CFUs per fly, assessed using LB and BHI plates, respectively. Thus, the composition of cultured gut bacteria differed significantly between OR9 and the rest of the OR populations.

To identify bacterial species selectively present in the OR9 versus the control OR3 population, we isolated all morphologically distinct colonies we could find in each population upon plating on LB and BHI media. The OR9 population contained seven morphologically distinct colonies, two of which were resistant to rifampicin, as opposed to the OR3 population that contained five morphologically distinct colonies, none of which could grow on rifampicin plates. We then performed colony PCR by extracting genomic DNA from single colonies of each morphological type, amplifying and sequencing PCR fragments corresponding to the 16S rRNA gene using pan-bacterial primers. The BLAST search of the sequences revealed five rifampicin-sensitive bacterial species, common to both OR9 and OR3, namely, *Staphylococcus xylosus*, *Staphylococcus homini*, *Pseudomonas putida*, *Bacillus mycoides* and *Paenibacillus pabuli* and two rifampicin-resistant strains exclusive to OR9, *E. haemoperoxidus* and *S. arlettae* (Figure 1D). To assess the impact of rifampicin-resistant strains on gut motility, germ-free OR9 and OR3 adult female flies were fed with *E. haemoperoxidus*, *S. arlettae* and *P. aeruginosa* (PA14) either alone or in various combinations, at equal concentrations of each bacterial strain. As shown in Figure 1E, the defecation rate was not significantly different between germ-free OR9 and OR3 female flies under any treatment, confirming that the two fly populations remained genetically comparable. However, the defecation rate significantly increased upon ingestion of *E. haemoperoxidus* either alone or in a triple combination with *S. arlettae* and PA14. Thus, ingestion of PA14 by flies bearing microbiota, as well as ingestion of *E. haemoperoxidus* by germ free flies can increase fly gut motility.

### 3.2. Water-Only and Ethanol-Containing Food Reduce Lifespan and Defecation Rate in Female Adults 

**Figure 2:**
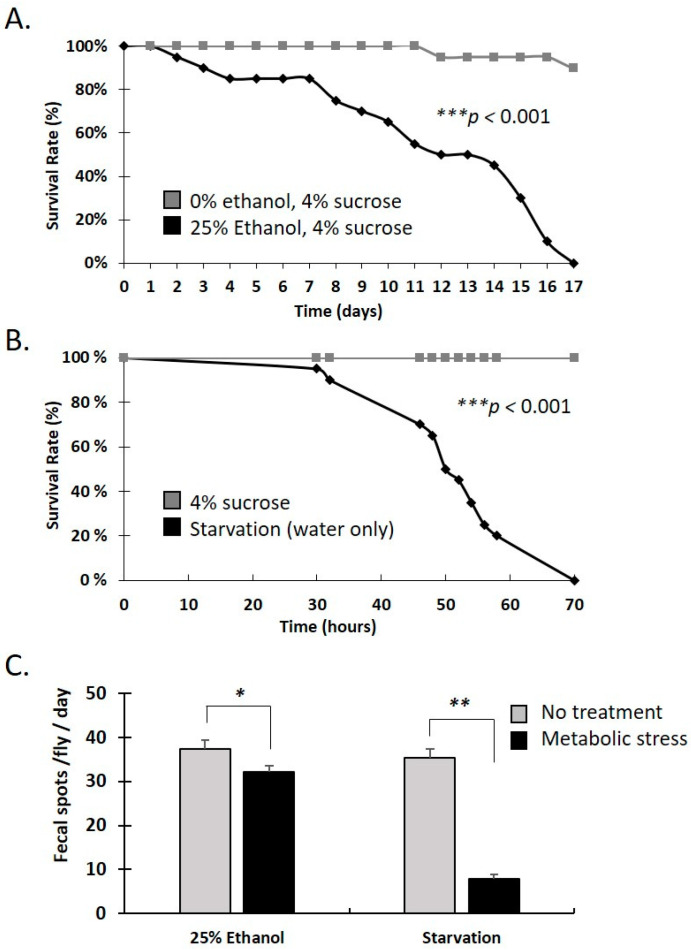
Starvation and dietary ethanol reduce *Drosophila* gut motility and lifespan. (**A**) Kaplan–Meyer survival curves of Oregon-R female flies, fed with either a 4% sucrose control diet (grey line) or 4% sucrose plus 25% ethanol (black line) (**B**) Survival curves of Oregon-R female flies, fed with either 4% sucrose (grey line) or starved, given only water (black line). For all experiments, *n* = 120 flies per condition. Statistical analysis was performed using the log-rank test. Statistical significance was observed in all cases (*** *p* < 0.001). (**C**) Fecal spots per fly per day upon feeding on 4% sucrose with and without 25% ethanol or upon water only starvation. Each column represents the mean value of six biological replicates. Error bars represent standard deviation of the mean. Statistical analysis was performed using the Mann–Whitney U test. Statistical significance indicated as * *p* < 0.05 or ** *p* < 0.01.

Starvation, ethanol consumption and other dietary stressors can impose long-lasting metabolic changes [33]. To assess the impact of dietary stresses, we measured fly survival rate over time upon ethanol consumption and water-only food. As shown in Figure 2A, flies consuming fly food containing 25% ethanol started dying at 2 days, reaching 50% mortality by 12 days and 100% mortality by 17 days (Figure 2A). 

On the other hand, water-only consuming flies started dying at around 30 h, reaching 50% mortality by 50 h and 100% mortality by 70 h (Figure 2B). Thus, water-only consumption exerted a much more severe stress on flies compared to the 25% ethanol containing diet.

To assess the effect of dietary stress on gut motility, fly defecation rate was measured following the 25% ethanol, 4% sucrose diet vs. 4% sucrose only as a control. Similarly, starvation (water-only) was compared to feeding on a 4% sucrose diet. As shown in Figure 2C, the 25% ethanol diet reduced defecation rate by ~14%, whereas water-only starvation by ~78%. Assessment for both treatments was performed at 24 h, a time point preceding the initiation of fly mortality under any of the treatments (Figure 2A,B). Therefore, water-only, and ethanol-containing food, reduce defecation rate within hours, analogous to their later impact on fly survival.

### 3.3. Genome Wide Analysis Reveals the Genetic Basis of Female Drosophila Defecation 

**Figure 3:**
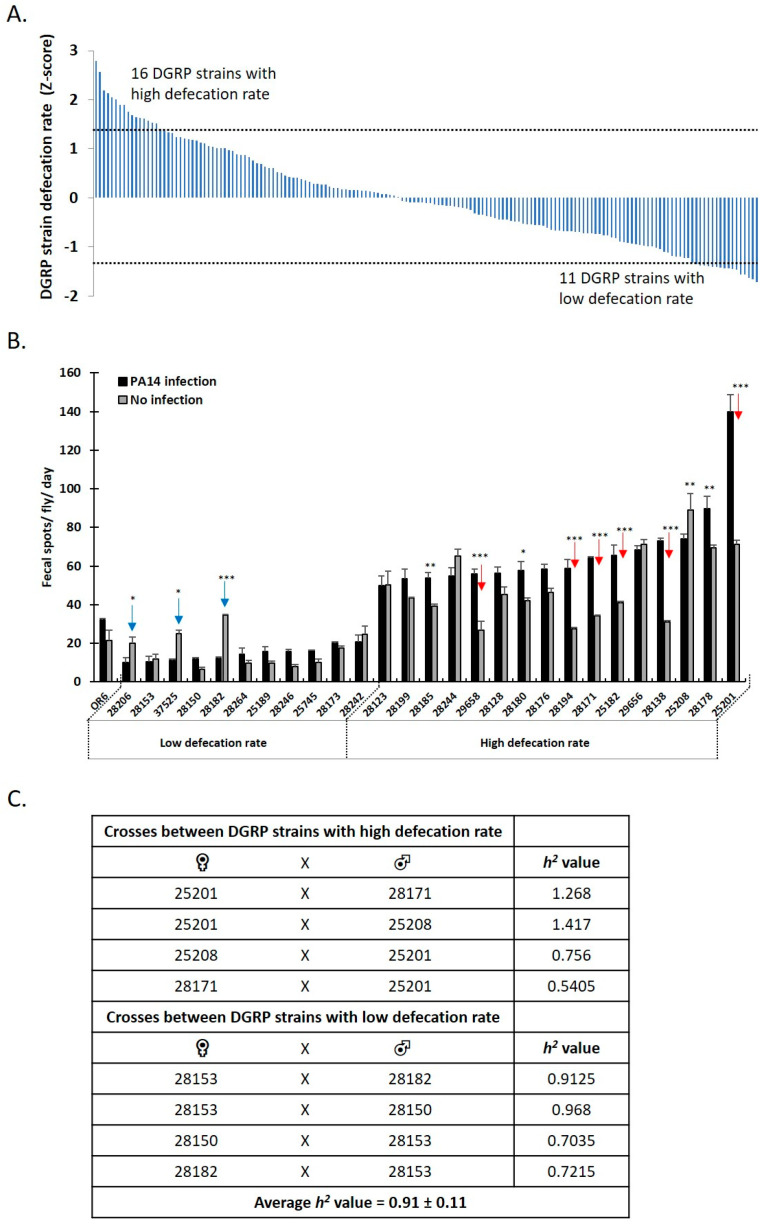
Phenotypic ranking and additive genetic basis of defecation rate of 150 DGRP strains. (**A**) Z-score analysis of the defecation rate of 150 DGRP strains, upon oral ingestion of P. aeruginosa. (**B**) Validation of defecation rate of 27 DGRP strains deviating by ≥1.25 SDs above or below the mean of the Z-score analysis, upon and without PA14 infection. Vertical red and blue arrows indicate a ≥2-fold change in the defecation rate, upon PA14 ingestion, in 6 out of 16 strains exhibiting extremely enhanced defecation rates and in 3 out of 11 strains exhibiting extremely reduced defecation rates, respectively. Each column represents the mean value of six biological replicates. Error bars represent standard deviation of the mean. Statistical analysis was performed using the Mann–Whitney U test. Statistical significance indicated as * *p* < 0.05 or ** *p* < 0.01, *** *p* < 0.001. (**C**) Narrow sense heritability ($h^{2}$) of the defecation rate was calculated based on crossing schemes between DGRP strains displaying high defecation rates (25201, 28171 and 25208) or low defecation rates (28182, 28150 and 28153). The standard error for the calculation of $h^{2}$ was 0.11.

Previous work in *Drosophila* has shown that genetic variability results in markedly different responses to environmental stresses, with some fly strains exhibiting poor homeostatic ability and metabolism control, while others remain relatively stable across environments [33]. To gain further insight into the impact of genetic background on fly defecation rate, 150 wild-type inbred lines of the Drosophila Genetic Reference Panel (DGRP) [29] were screened upon ingestion of an infection mix containing PA14. The Z-score analysis underscored the phenotypic continuity of the trait, which ranged between 4.28 standard deviations of the mean (SDs) (Figure 3A).

Strains deviating by ≥1.25 SDs above or below the mean were selected, corresponding to 16 strains exhibiting the highest and 11 strains exhibiting the lowest defecation rates (Figure 3A). Six out of the 16 strains displaying high defecation rates (red arrows in Figure 3B) and three out of the 11 strains displaying low defecation rates (blue arrows in Figure 3B) exhibited ≥2-fold change in the defecation rate due to infection. Overall, 13 out of the 27 strains displaying high or low defecation rates exhibited a significant change in the defecation rate due to infection (asterisks in Figure 3B). These results clearly indicate that genetic factors and intestinal infection can have a significant contribution to the determination of the defecation rate.

To quantify the contribution of genetic factors in our defecation rate assay, we determined the narrow sense heritability ($h^{2}$), that is, the fraction of phenotypic variation attributed to the additive effects of genes [34]. As shown in Figure 3C, a total of eight crossing schemes were performed: four between DGRP strains displaying high defecation rates (25201, 28171 and 25208) and four between DGRP strains displaying low defecation rates (28182, 28150 and 28153). Both the parents’ and offspring’s defecation rates were then determined and used to calculate the $h^{2}$ value for each crossing scheme (see Methods). As shown in Figure 3C, $h^{2}$ values ranged between 0.54 and 1.27, while the $h^{2}$ average value was 0.91 *±* 0.11 (standard error). This indicates that even though single crossing scheme calculations of $h^{2}$ vary from one to another, the average phenotypic variation is close to 1, and, thus, attributable to variations in additive gene alleles. This agrees with previous work showing that a significant component of the genetic variance of a trait is additive, when considering populations with many loci at extreme allele frequencies [34].

We then proceeded with a genome wide association study (GWAS) to pinpoint specific genes associated with defecation rate, using the DGRP GWAS Webtool (http://dgrp2.gnets.ncsu.edu/, accessed on 1 January 2023). This analysis led to the identification of 17 genes, many of which were linked to more than one SNP (Table 1).

Fourteen out of the total 17 GWAS-identified genes (excluding the basement membrane forming genes *vkg* and *pxn* and the miRNA gene *mir-971*) were further functionally assessed as genetic determinants of the defecation rate.

### 3.4. Defecation Rate Is Controlled in Female Midgut EEs and Neurons by GWAS-Identified and Hormone-Encoding Genes

**Figure 4:**
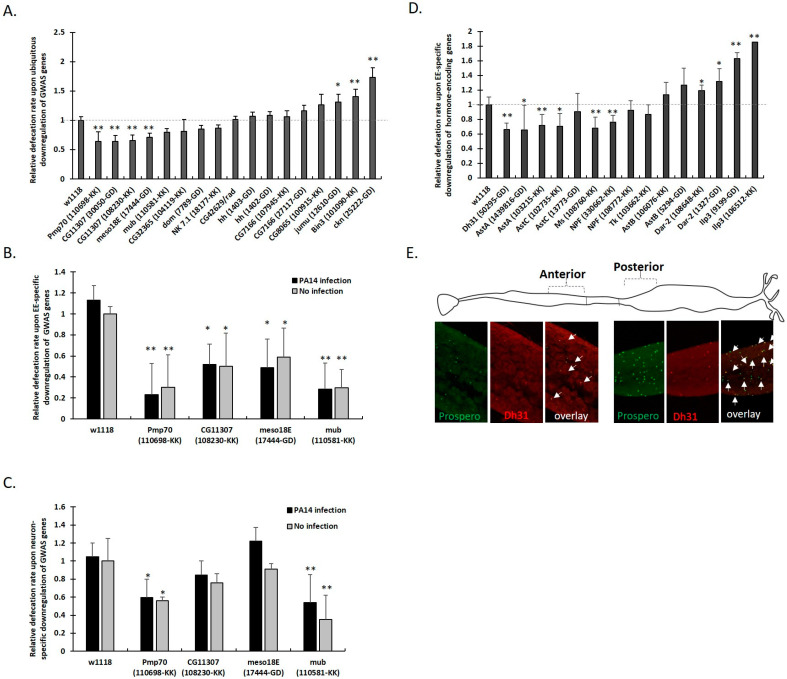
GWAS-identified and hormone gene downregulation either ubiquitously or specifically in EEs or neurons affects fly defecation rate. Fecal spots per fly per day upon (**A**) ubiquitous (via *act-Gal4 UAS-RNAi*), (**B**) EE-specific (via *prosV1-Gal4 UAS-RNAi*) and (**C**) pan-neuron-specific (via *elav-Gal4 UAS-RNAi*) downregulation of indicated GWAS-identified genes, divided by that of the uninfected progeny of Gal4 crossed to *w^1118^*. (**D**) Fecal spots per uninfected fly per day upon EE-specific (via *prosV1-Gal4 UAS-RNAi*) downregulation of hormone genes, divided by that of the progeny of Gal4 crossed to *w^1118^* flies. Each column represents the mean value of six biological replicates. Error bars represent standard deviation of the mean. Statistical significance using the Mann–Whitney U test indicated as * *p* < 0.05 or ** *p* < 0.01. (**E**) Schematic of the *Drosophila* midgut, indicating the anterior and posterior regions and the expression of *Dh31-Gal4 UAS-mCherry-NLS* reporter (red) that colocalizes with anti-Prospero antibody staining of EEs (green), indicated by white arrows.

To functionally validate genes identified through GWAS analysis, we crossed the corresponding UAS-RNAi lines—obtained from the Vienna Drosophila Research Center (VDRC)—with the ubiquitous Gal4 driver line, act-Gal4. Defecation rates were evaluated for each offspring and mean values were divided by those of the control (act-Gal4 crossed with *w^1118^*). As shown in Figure 4A, ubiquitous downregulation of *Pmp70*, *CG11307*, *meso18E* and *mub* decreased defecation rates, while that of *Bin3*, *ckn* and *jumu* increased defecation rates (Figure 4A).

To determine whether the inducers of defecation rate, *Pmp70*, *CG11307*, *meso18E* and *mub*, have a more direct involvement in the regulation of the gut-brain axis, we selectively downregulated them in either the midgut EEs via prosV1-Gal4 or in all neurons via elav-Gal4. Selective downregulation of all four genes in EEs (Figure 4B) and of *Pmp70* and *mub* in all neurons (Figure 4C) decreased the defecation rate, irrespective of the infection status, suggesting that these genes are critical for hormonal signaling along the gut-brain axis.

To delineate the communication between midgut and neurons, we assessed the role of nine *Drosophila* hormone related genes expressed in the *Drosophila* neurons and midgut EEs, namely, *AstA*, *AstB*, *AstC*, *Dh31*, *NPF*, *Dar2*, *Tk*, *Ms* and *insulin-like peptide 3 (ilp3)* [21,23,35,36,37]. Downregulation of *AstA*, *AstC*, *Dh31*, *NPF*, and *Ms* in EEs led to decreased defecation rates, while downregulation of *Dar2* and *ilp3* led to increased defecation rates (Figure 4D and Figure S1A).

Co-localization of GFP with anti-prospero staining of *Dh31-Gal4 UAS-gfp* flies confirmed that Dh31 is expressed mainly in a subset of posterior midgut EEs and less so in a subset of anterior EEs (Figure 4E) [14,15,38]. The reduced defecation rate resulting from *Dh31* downregulation in EEs (Supplementary Figure S1A) correlates with the reduced defecation rate observed upon downregulation of *Dh31 receptor* (*Dh31R*) in the visceral muscle (VM) using the 24B-Gal4 (Supplementary Figure S1B). Moreover, downregulation of either Dh31 or Dh31R in neurons, also led to reduced defecation rates (Supplementary Figure S1C). In conclusion: (a) expression of *AstA*, *AstC*, *NPF*, *Ms* and * Dh31* in EEs induces defecation rate, (b) *Dh31* signals from midgut EEs to its receptor in the VM, increasing the defecation rate, and presumably midgut muscle contractions, as previously reported [14], and (c), *Dh31*-expressing neurons can induce defecation rate via signaling to *Dh31R*-expressing neurons.

### 3.5. Higher Expression of NPF and CG11307 in DGRP Lines Displaying High Defecation Rates

**Figure 5:**
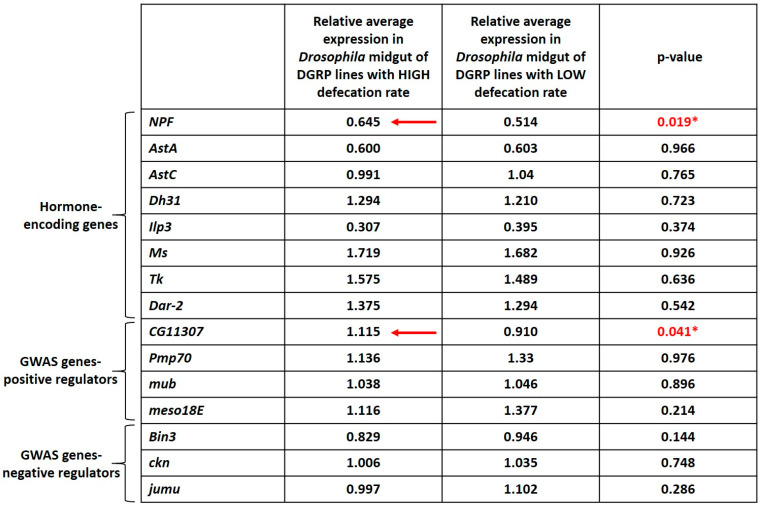
High defecation rate DGRP lines exhibit higher midgut expression of *NPF* and *CG11307*, indicated by horizontal red arrows. Midgut expression of hormone encoding genes (*AstA*, *AstC*, *Dh31*, *Ilp3*, *Ms*, *Tk*, *Dar-2*) and GWAS-identified genes considered either positive regulators of the defecation rate, (*Pmp70*, *mub*, *meso18E*) or negative regulators (*Bin3*, *ckn* and *jumu*), in eight DGRP lines displaying high (25208, 28128, 25201, 28180, 28171, 28123, 29658, 28244) versus eight DGRP lines displaying low (28206, 28182, 28153, 28246, 25745, 25189, 28242, 28150) defecation rates. For each of the 16 DGRP lines the mean expression value of six biological replicates per gene was calculated and normalized to that of the strain OR6, and then used to find the relative average expression per group of eight high and eight low defecation rate lines. Statistical analysis was performed using the Mann–Whitney U test. Statistical significance (*p* < 0.05) is indicated with asterisks and red font for *NPF* and *CG11307*.

To associate genes to defecation rate in an alternative way, we assessed midgut expression of seven GWAS-identified (4 positive regulators, *Pmp70*, *CG11307*, *mub*, *meso18E*, and 3 negative regulators, *Bin3*, *ckn*, *jumu*) and eight hormone-encoding genes (*AstA*, *AstC*, *Dh31*, *NPF*, *Dar2*, *Tk*, *Ms*, *ilp3*) comparing expression between a group of eight high and a group of eight low defecation rate DGRP strains. As shown in Figure 5, gene expression of NPF, was increased on average in DGRP lines displaying higher defecation rate.

This agrees with previous work, showing that NPF acts as a hunger signal, promoting wakefulness and adult feeding, which in turn may enhance gut motility and defecation rate [36]. Moreover, expression of *CG11307*, a novel gene identified through our GWAS, displayed a similar pattern, that is, a higher expression in the high defecation rate strains (Figure 5), further supporting the role of this gene in controlling defecation rate.

### 3.6. GWAS-Identified and Hormonal Genes Participate in a Defecation Rate Signaling Network

**Figure 6:**
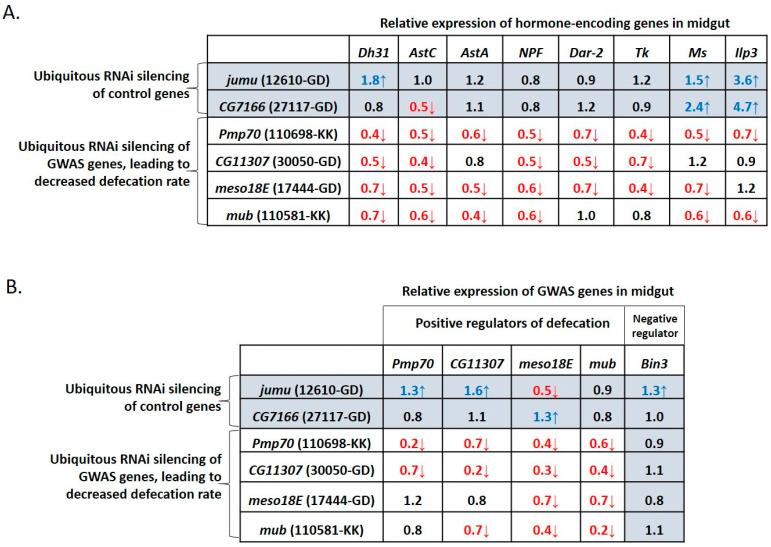
Midgut expression levels of hormone-encoding and GWAS genes upon ubiquitous downregulation of GWAS-identified positive regulator genes of the defecation rate. Relative expression levels of (**A**) hormones and (**B**) GWAS-identified genes associated with defecation rate, in the midgut of flies upon ubiquitous downregulation (via act-Gal4-UAS-RNAi) of positive regulators of the defecation rate, *Pmp70*, or *mub*, or *meso18E*, or *CG11307*, normalized to flies prepared from Gal4 lines crossed to *w^1118^*. Ubiquitous downregulation of either *jumu* or *CG7166* was used as a control. Values indicated in red represent a ≥0.3 decrease (red arrow pointing down), whereas values in blue represent a ≥0.3 increase (blue arrow pointing up) from the baseline value of 1. Each number represents the average of six values.

To assess the cross-regulation potential of defecation-controlling genes we assessed the expression pattern of the eight defecation-associated hormonal genes (*AstA*, *AstC*, *Dh31*, *NPF*, *Dar2*, *Tk*, *Ms*, *ilp3*) upon ubiquitous downregulation of the GWAS-identified genes, *Pmp70*, *CG11307*, *meso18E* and *mub*. Downregulation of *CG7166* and *jumu*, served as independent controls, given that these genes did not act as positive regulators of the defecation rate under our experimental conditions (Figure 4A). Ubiquitous silencing of *Pmp70* led to a 30–50% reduction in the expression levels of all hormones examined (red font in Figure 6A).

Ubiquitous downregulation of *meso18E*, *mub* and *CG11307* led to a 30–70% reduction in the expression of most hormone genes examined, that is, seven, six and five out of eight, respectively. In contrast, downregulation of the control genes, *CG7166 *and *jumu*, showed no evidence of such a trend, since it led to reduced expression of one and zero genes, respectively, and induced expression of two and three out of eight hormonal genes, respectively (blue font in Figure 6A).

To determine whether the GWAS-identified genes, *Pmp70*, *CG11307*, *meso18E* and *mub*, could regulate each other, we examined how ubiquitous downregulation of each gene affected expression of the rest. Downregulation of *Bin3*, a GWAS-identified gene, served as a control, given that this gene acted as a negative regulator of the defecation rate under our experimental conditions (Figure 4A). Ubiquitous silencing of *Pmp70* and *CG11307* led to a 30–70% reduction in the expression levels of all GWAS-identified inducers of defecation (red font in Figure 6B) but had no obvious effect on the expression of *Bin3*, the negative regulator of defecation rate. Ubiquitous silencing of *meso18E* and *mub* led to a 30–60% decrease in the expression of two and three out of six GWAS-identified genes, respectively, all of which are potential positive regulators of the defecation rate. Importantly, silencing of the control genes *CG7166* and *jumu* showed no evidence of such a trend, leading instead to increased expression of one and three out of six GWAS-identified genes, respectively, including that of the negative regulator gene, *Bin3* (blue font in Figure 6B). Thus, GWAS-identified inducers of defecation participate in a gene network that involves their cross-regulation and the regulation of hormonal genes.

### 3.7. GWAS-Identified Inducers of Defecation Induce Dh31 Expression in the Midgut and Reduce it in the Head

**Figure 7:**
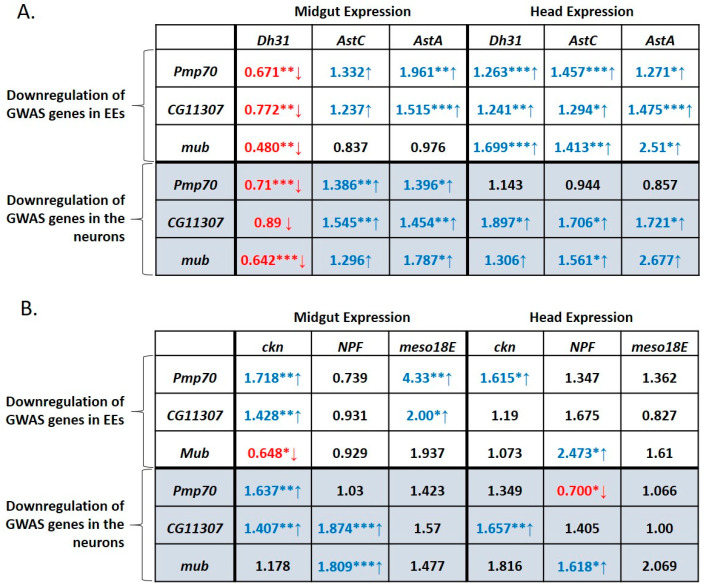
Downregulation of GWAS genes, *mub*, *CG11307* and *Pmp70*, in EEs or neurons consistently reduces *Dh31* and increases *AstC* and *AstA* expression in the midgut, while *Dh31*, *AstC* and *AstA* expression are all increased in the head. Relative expression levels of (**A**) hormone-encoding genes *Dh31*, *AstC* and *AstA* and (**B**) GWAS-identified genes *ckn*, NPF and *meso18E* in the midgut and head of flies upon EE-specific (via prosV1-Gal4-UAS-RNAi) or pan-neuronal (via elav-Gal4-UAS-RNAi) downregulation of *Pmp70*, *mub* or *CG11307*, normalized to flies prepared from Gal4 lines crossed to *w^1118^*. Values in red font accompanied by a red arrow pointing down represent statistically significant decrease. Values in blue font accompanied by a blue arrow pointing up represent statistically significant increase. Each number represents the average of 12 values. Statistical analysis was performed using the *t*-test. Statistical significance indicated as * *p* < 0.05, ** *p* < 0.01 or *** *p* < 0.001.

To delineate key gene network factors involved in regulating the defecation rate via the gut-brain axis, we downregulated each of the GWAS-identified inducers of defecation, *Pmp70*, *CG11307* and *mub*, in the midgut EEs and neurons, while assessing the expression of hormonal genes in dissected midguts and heads. As shown in Figure 7, a clear pattern emerged, involving three hormonal genes, *Dh31*, *AstA* and *AstC*. Either EE- or neuron-targeted downregulation of *Pmp70*, *CG11307* or *mub* led to decreased *Dh31* expression in the midgut and increased *Dh31* expression in the head.

Concomitantly, the expression of *AstC* and *AstA* was significantly increased in both the midgut and the head, irrespective of which cells (EEs or neurons) were targeted for downregulation of *Pmp70*, *CG11307* or *mub*. Thus, there is a crosstalk between the midgut and the head implicating the GWAS-identified positive regulators of defecation and the hormonal genes, *Dh31*, *AstA* and *AstC*. Notably, both EE- and neuron-expressed *Pmp70*, *CG11307* and *mub*, regulate *Dh31* expression positively in the midgut and negatively in the head.

In addition, we examined the expression of two potential positive regulators of gut motility, namely *NPF* and *meso18E*, as the former was overexpressed in DGRP strains displaying high defecation rate (Figure 5A) whereas ubiquitous silencing of both in EEs led to decreased defecation rate (Figure 4B,D), as well as *ckn*, a potential negative regulator, ubiquitous silencing of which led to increased defecation rate (Figure 4A). The expression of *NPF* remained largely unaffected in the midgut and head upon silencing of *Pmp70*, *CG11307* or *mub* in EEs (Figure 7B). With the notable exception of decreased *NPF* expression in the head upon *Pmp70* downregulation in the neurons, an inhibitory crosstalk between positive regulators by *CG11307* and *mub* was noticed upon downregulation in neurons (Figure 7B).

The expression of *meso18E* remained unaffected in the head, but it was increased in the midgut upon *Pmp70* and *CG11307* (and potentially *mub*) downregulation in EEs (Figure 7B), revealing an inhibitory crosstalk between these positive regulators in the midgut. Lastly, expression of the negative regulator, *ckn*, in the midgut and/or head was increased upon downregulation of *Pmp70* and *CG11307* in EEs or neurons (Figure 7B). This points towards the existence of another crosstalk between the midgut and the head implicating the GWAS-identified genes *pmp70* and *CG11307* and the negative regulator *ckn*.

### 3.8. Water-Only Consumption Reduces Dh31, AstC, AstA and Pmp70 Expression in the Midgut and Reduced Expression of meso18E in the Head

**Figure 8:**
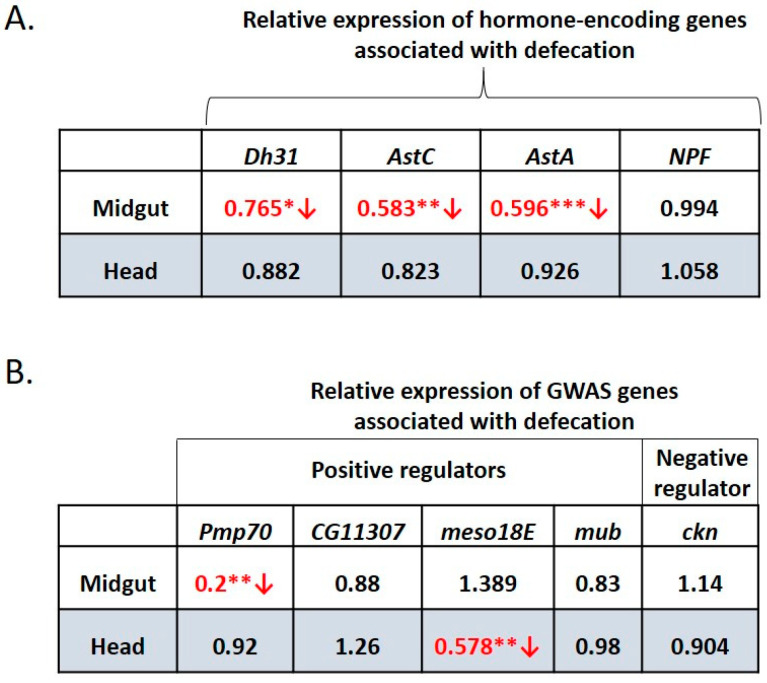
Starvation reduces expression of hormonal genes, *Dh31*, *AstC* and *AstA*, and positive regulators, *Pmp70* in the midgut, and *meso18E* in the head. Relative expression of (**A**) hormone-encoding genes *Dh31*, *AstA*, *AstC* and *NPF* and (**B**) GWAS-identified positive (*Pmp70*, *CG11307*, *meso18E* and *mub*) and negative (*ckn*) regulators of the defecation rate, in midgut and head, upon starvation vs. feeding on 4% sucrose of female OR6 adults. Values indicated in red accompanied by a red arrow pointing down represent statistically significant decrease in the relative hormone gene expression. Each number represents the average of twelve values. Statistical significance using the *t*-test is indicated as * *p* < 0.05, ** *p* < 0.01 or *** *p* < 0.001.

Next, we sought to assess the impact of a dietary stressor on the expression of the hormonal genes, *NPF*, *Dh31*, *AstA* and *AstC*, and of the GWAS-identified genes, *Pmp70*, *CG11307*, *meso18E*, *mub* and *ckn*, in the midguts and heads of wild type female adults at 24 h of water-only consumption (starvation). As shown in Figure 8A, starvation led to a statistically significant decrease in the expression of *Dh31*, *AstA*, *AstC* and *Pmp70* in the midgut but not the head.

Moreover, starvation also led to a statistically significant decrease in the expression of *meso18E* in the head (Figure 8B), further demonstrating that these GWAS-identified genes are implicated in the defecation rate.

In contrast, expression of *NPF*, was not significantly altered, in either organ, upon starvation. Similarly, expression of *CG11307* and *mub* was not significantly altered, upon starvation, which however does not exclude their involvement in defecation under different stress conditions. Other stressors or homeostatic states may induce or involve different regulators of defecation.

### 3.9. Downregulation of Dh31 in the Midgut EEs or in Neurons Reduces Dh31 Expression in Both the Midgut and Head

**Figure 9:**
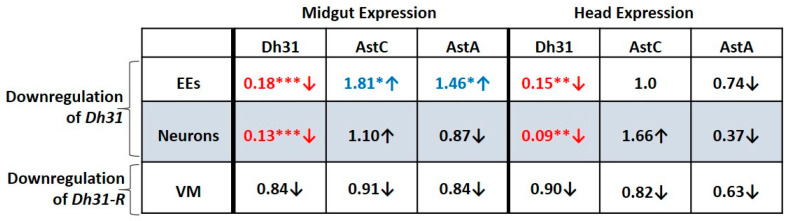
Downregulation of *Dh31* in EEs or neurons reduces *Dh31* expression in both the midgut and the head, while downregulation of *Dh31* in EEs increases *AstC* and *AstA* expression in the midgut. Relative expression levels of hormone-encoding genes *Dh31*, *AstA* and *AstC* in the midgut and the head upon EE-specific (via prosV1-Gal4-UAS-RNAi) or pan-neuron-specific (via elav-Gal4-UAS-RNAi) downregulation of *Dh31* or VM-specific (via 24B-Gal4-UAS-RNAi) downregulation of *Dh31-R*, normalized to flies prepared from Gal4 lines crossed to *w^1118^*. Values indicated in red and blue represent statistically significant decrease (red arrow pointing down) and increase (blue arrow pointing up) respectively, of the relative hormone gene expression. Each number represents the average of 12 values. Statistical analysis was performed using the *t*-test. Statistical significance indicated as * *p* < 0.05, ** *p* < 0.01 or *** *p* < 0.001.

To determine whether *Dh31* regulates its own expression and that of the associated peptide hormone genes, *AstA* and *AstC*, along the midgut-brain axis, we downregulated *Dh31* in EEs using the prosV1-Gal4 and in all neurons using the elav-Gal4. We also downregulated its receptor, *Dh31-R*, in the visceral muscle using the 24B-Gal4. We then assessed the expression of *Dh31*, *AstA* and *AstC* in dissected midguts and heads. As shown in Figure 9, downregulation of *Dh31* in either EEs or neurons led to reduced *Dh31* expression in both the midgut and the head.

Moreover, downregulation of *Dh31* in EEs increased *AstA* and *AstC* expression in the midgut but not the head, whereas downregulation of *Dh31* in neurons did not significantly affect *AstA* and *AstC* expression in the midgut or head. Downregulation of *Dh31-R* in the visceral muscle did not significantly alter the expression of *Dh31*, *AstA* or *AstC* in the midgut or head, indicating that Dh31-Dh31R signaling in the VM does not affect the expression of these hormone peptide genes. Thus, *Dh31* autoregulates itself within and between cells of the midgut and head.

## 4. Discussion

Gastrointestinal motility is a polygenic trait critical for energy homeostasis, host-microbiota interaction, and overall health. It is influenced by mechanistically complex genetic and environmental factors. Here, we addressed the contribution of environmental and genetic factors on gut motility using a customized *Drosophila* defecation assay amenable to microbial infection and dietary treatments.

We showed that wild type fly populations, of the same genotype, reared in parallel in the lab for a year, bear gut bacterial strains belonging to the species *S. xylosus*, *S. homini*, *P. putida*, *B. mycoides* and *P. pabuli* that are sensitive to the antibiotic rifampicin, as well as two rifampicin-resistant strains, namely *E. haemoperoxidus* and *S. arlettae*. Interestingly, the defecation rate is comparable and similarly induced upon oral bacterial infection, when these genetically identical populations, reared apart, are rendered germ-free. This agrees with previous studies showing that, even though some bacteria are frequent colonizers of the *Drosophila* gut, fly stock microbiota can differ greatly between laboratories and even between stocks raised independently within the same laboratory [39,40]. Similarly, the human microbiota is highly house-dependent, as it is more comparable among genetically unrelated individuals who share a household, than among relatives who do not have a history of household sharing [41].

Furthermore, we provide evidence of microbiota as cofounding factors, affecting the defecation rate. We identified *E. haemoperoxidus*, as a fly microbiota isolate, the ingestion of which sufficed to increase defecation rate in germ-free flies. On the other hand, ingestion of *P. aeruginosa*, a human opportunistic pathogen increased the defecation rate of gnotobiotic flies associated with *E. haemoperoxidus* and *S. arlettae*, but not of germ-free flies, indicating that *P. aeruginosa* requires intestinal microbiota to induce gut motility. Previous work showed that *Dh31* expression in the fly midgut EEs is associated with strong visceral muscle contractions following the ingestion of harmful bacteria, a mechanism by which flies may propel quick expulsion of pathogens from the fly gut [14]. Therefore, metabolites or virulent factors produced by *E. haemoperoxidus* and *P. aeruginosa* may stimulate midgut EEs to release Dh31, which in turn increases defecation rate.

Moreover, water-only starvation and dietary ethanol impose significant stress and reduce defecation rate, analogously to their impact on fly survival. In our study, water-only consumption was compared to a 4% sucrose diet, both of which do not provide any solid residues, and agrees with previous work showing that starvation lowers the defecation rate long before the gut is emptied [42]. Starvation has been shown to influence various aspects of fly behavior, including foraging, feeding, sleep, agility, courtship, mating, reproduction, and nutrient sensing, thus emphasizing the importance of signaling along the gut-brain axis [42,43,44,45,46]. Mechanisms regulating the gut-brain axis communication involve a diverse array of neuropeptide hormones secreted primarily by midgut EEs and the brain [37,47]. Here, we show that starvation reduces the expression of *Dh31*, *AstC* and *AstA* in the fly midgut, but not in the head. By reducing *Dh31* expression in the fly midgut starvation may in turn reduce midgut muscle contractions [14] and the rate of defecation. Estimation of heritability serves as a useful tool across a range of disciplines, from evolutionary biology to agriculture to human medicine [48]. It has been applied to the estimation of genetic variation in human behavioral phenotypes, such as IQ, and the risk for many disorders, such as schizophrenia, autism, and attention deficit hyperactivity disorder [49,50,51]. Genetic variation also determines gut motility. Defecation rate studies in *C. elegans* and rats, as well as four small and one large study of ~168,000 humans from the UK BioBank reported heritability in stool frequency [52,53,54]. Accordingly, we show that the naturally occurring genetic variation differentially affects the defecation rate in DGRP strains, identifying 16 strains displaying high and 11 strains displaying low defecation rates. We found the narrow-sense heritability ($h^{2}$) in our customized assay to equal 0.91, indicating that the genetic variance in defecation rate is mostly additive, which is typical of a polygenic trait. Nevertheless, heritability is a population parameter. It depends on population-specific factors, such as allele frequencies, the effects of gene variants, and variation due to environmental factors [48]. It does not necessarily predict the value of heritability in other populations or other species. However, the heritability of many traits depends mainly on additive genetic variance across populations and species [34].

Peptide hormones have been previously associated with feeding behavior, metabolism, and energy homeostasis. For example, secretion of Dh31, Ast A and AstC suppresses feeding [22,24,55], NPF regulates food choice behavior [17,18], Tk confers both excitatory and inhibitory effects on gut motility [26], and Ms relaxes crop muscles, to allow expansion and increased food intake [10]. Herein we show that downregulation of *AstA*, *AstC*, *Dh31*, *NPF*, *Tk* or *Ms* in midgut EEs lead to decreased defecation rates. This result complements previous work showing that loss of *Dh31* expression from the larval midgut EEs impairs peristalsis [13]. In contrast downregulation of *Dar2* or *ilp3* increased the defecation rate. Interestingly, the production of some of these peptides in the midgut EEs is very low compared for example to the expression of *Dh31* and *NPF* [37]. This is also true at the gene expression level so that, for example, *ilp3* and *Ms* expression, while more abundant in the midgut EEs compared to other midgut cell types, it is a few orders of magnitude lower than the expression of *NPF* and *Dh31* [38]. However, we find that the downregulation of lowly expressed peptide hormones, such as *Ilp3* and *Ms*, can have an impact on defecation rate comparable to that of *Dh31* and *NPF*.

We also positively correlated expression of *NPF* and *CG11307* (a newly identified GWAS gene) with the defecation rate of genetically disparate DGRP strains, reinforcing the regulatory role of these genes. This is in line, with previous work showing that NPF acts as a hunger signal promoting wakefulness and adult feeding [36], which presumably enhances gut motility. Accordingly, we show that starvation decreased *Dh31*, *AstC* and *AstA* expression in the midgut, while *NPF* expression remained unaltered upon starvation. Different treatments and stimuli may activate alternative hormone peptides and cell types leading to differential regulation of gut motility. For example, NPF does not specifically influence total food intake, but may rather regulate food choice behavior [17,18].

Our GWA analysis identified 17 genes associated with defecation rate. Functional assessment enabled focusing on four of these genes, namely *Pmp70*, *CG11307*, *meso18E* and *mub*, as inducers of defecation rate; *CG11307* is not phenotypically annotated; *Pmp70* is orthologous to human *ABCD3* (ATP binding cassette subfamily D member 3) and is predicted to be involved in fatty acid catabolic processes, long-chain fatty acid import into the peroxisome and peroxisome organization [56,57]; *meso18E* is involved in mesoderm development, including development of the visceral muscle primordium [58,59]; *mub* is involved in thermosensory and satiety signals controlling food-seeking behavior [60,61,62]. Ubiquitous, independent downregulation of *Pmp70*, *CG11307*, *meso18E* and *mub* reduced the defecation rate, while ubiquitous, independent downregulation of *jumu*, *ckn* and *Bin3* increased the defecation rate. Similarly, targeted downregulation of *Pmp70*, *CG11307*, *meso18E* and *mub* in midgut EEs reduced the defecation rate, indicating that these genes act tissue- and cell-specifically, in the midgut EEs promoting defecation.

**Figure 10:**
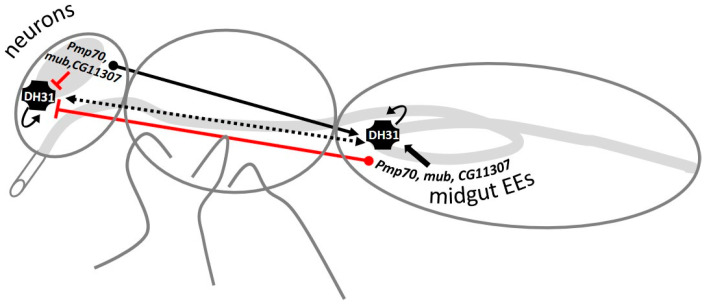
*Pmp70*, *mub* and *CG11307*, control *Dh31* positively in the midgut and negatively in the head, irrespective of the tissue being targeted for downregulation (midgut EEs or neurons). Proposed mechanism depicting the communication between GWAS-identified genes and *Dh31* along the gut-brain axis. While a positive regulator of defecation rate, *Dh31* is induced in the midgut via *Pmp70*, *mub*, *CG11307* expression in EEs or neurons. Interestingly, *Dh31* is inhibited in the head via *Pmp70*, *mub*, *CG11307* expression in EEs or neurons. However, *Dh31* positively regulates itself, locally and remotely, in the midgut and head tissues, when expressed in either midgut EEs or neurons. Red lines on the diagram represent negative regulation while the black arrows represent positive regulation of defecation rate.

We also found a role for the GWAS-identified inducers of defecation, *Pmp70*, *CG11307*, *meso18E* and *mub*, in altering the expression of the peptide hormone genes, *Dh31*, *AstC* and *AstA*, in the *Drosophila* midgut and head. These GWAS-identified genes are expressed in both the midgut EEs and in neurons, yet they have distinct roles depending on their site of action. They activate *Dh31* in the midgut, but inhibit its expression in the head, irrespective of their own expression site of origin (Figure 10).

Despite the tissue specificity of the four GWAS-identified inducers of defecation, *Dh31* functions as an activator of defecation rate, regardless of the cell type in which it is expressed (midgut EEs or neurons). This may be partly explained by recent work showing that feeding increases the production and release of DH31 from midgut EEs into the hemolymph and in turn excites two populations of Dh31-R expressing neurons in the brain, one inhibiting feeding through Ast-C and another promoting courtship through corazonin [55]. It is possible that the newly identified *Dh31*-controlling genes activate the Dh31-R expressing neurons in the fly brain, which may inhibit feeding and thus reduce gut motility [34,35,36,37].

## Figures and Tables

**Table 1:** Defecation rate associated genes identified by GWAS analysis. Genes associated with defecation rate identified by phenotypic analysis of 150 DGRP strains and the DGRP GWAS Webtool (http://dgrp2.gnets.ncsu.edu/) (accessed throughout 2017–2022). Commonly occurring polymorphisms (SNPs) associated with 17 genes and their function and midgut expression are shown.

Gene Affected (FlyBase ID, Name)	Associated Variant ID (SNPs)	Variant Hits per Gene	Gene Function and Midgut Expression (Flybase and FlyGutSeq)
FBgn0016075, *vkg*	2L_5025290_SNP 2L_5025252_SNP 2L_5025248_SNP	3	Subunit of Collagen IV, a major component of basement membranes. Anterior midgut visceral muscle expression and enteroendocrine cell induction by infection.
FBgn0261388, *CG42629*	X_12955420_SNP X_12955430_SNP	2	radish (rad) encodes a protein involved in anesthesia-resistant memory, heart contraction regulation, and small GTPase mediated signal transduction.
FBgn0262222, *mir-971*	X_12955420_SNP X_12955430_SNP	2	Micro-RNA gene (within the rad locus) of unknown function. Enterocyte and visceral muscle expression.
FBgn0020306, *dom*	2R_17221075_SNP 2R_17220640_SNP 2R_17220646_SNP	3	Tip60 acetyltransferase complex and functions in the exchange of histone His2Av. Uniform expression and stem cell induction upon infection.
FBgn0031069, *Pmp70*	X_19647084_SNP	1	ATPase-coupled peroxisomal transmembrane transporter of long-chain fatty acids. High enteroendocrine cell and visceral muscle expression.
FBgn0037106, *CG11307*	3L_21612306_SNP 3L_21612307_SNP	2	Unknown function. Enteroendocrine cell expression.
FBgn0037107, *CG7166*	3L_21612306_SNP 3L_21612307_SNP	2	Predicted to be involved in homophilic cell adhesion. Enteroendocrine cell expression.
FBgn0262737, *mub*	3L_21916473_SNP 3L_21853239_SNP	2	Regulation of alternative mRNA splicing.
FBgn0033987, *ckn*	2R_10853015_SNP	1	Cell contact and tyrosine phosphatase signaling pathway. Enteroendocrine cell expression.
FBgn0024321, *NK7.1*	3R_10187071_SNP	1	Regulation of transcription by RNA polymerase II. Uniform expression.
FBgn0040089, *meso18E*	X_19604135_SNP	1	Visceral muscle development. Uniform expression and progenitor cell bias.
FBgn0004644, *hh*	3R_18968168_SNP	1	Morphogen. Uniform expression and enteroblast bias.
FBgn0016075, *CG8065*	3L_10208120_SNP	1	Unknown function. Enterocyte lineage expression.
FBgn00263144, *Bin3*	2R_2126406_SNP	1	mRNA translation inhibition. Uniform expression and enterocyte bias.
FBgn0052365, *CG32365*	3L_7873543_SNP	1	Unknown function. Uniform expression and progenitor cell bias.
FBgn0001316, *klar*	3L_461631_SNP	1	Organelle movement via microtubules. Uniform expression and enteroblast bias.
FBgn0011828, *pxn*	3L_2603619_SNP	1	Collagen IV cross-linking. Uniform expression, enteroendocrine cell induction by infection.

## Data Availability

Data are contained within the article or the Supplementary Material.

## Supplementary Materials

The following supporting information can be downloaded at https://www.mdpi.com/article/10.3390/metabo13020264/s1. Figure S1: Both downregulation of *Dh31* in EEs or neurons and downregulation of *Dh31*-receptor (Dh31-R) in Visceral Muscle (VM) or neurons reduces the defecation rate; Table S1: Gene specific qPCR primer sequences.

## References

[1] Kitazawa T., Kaiya H. (2019). Regulation of Gastrointestinal Motility by Motilin and Ghrelin in Vertebrates. Front. Endocrinol..

[2] Monteiro M.P., Batterham R.L. (2017). The Importance of the Gastrointestinal Tract in Controlling Food Intake and Regulating Energy Balance. Gastroenterology.

[3] Daniel N., Lécuyer E., Chassaing B. (2021). Host/Microbiota Interactions in Health and Diseases-Time for Mucosal Microbiology!. Mucosal Immunol..

[4] Iacob S., Iacob D.G., Luminos L.M. (2018). Intestinal Microbiota as a Host Defense Mechanism to Infectious Threats. Front. Microbiol..

[5] Rozé C. (1980). Neurohumoral Control of Gastrointestinal Motility. Reprod. Nutr. Dev..

[6] Daniel E., Tougas G., Allescher H.D., Vergara P., Fox-Threlkeld J.A. (1994). Mediators and Enteric Nerve Pathways Controlling Gastric Emptying. Dig. Dis. Sci..

[7] Travagli R.A., Anselmi L. (2016). Vagal Neurocircuitry and Its Influence on Gastric Motility. Nat. Rev. Gastroenterol. Hepatol..

[8] Boeckxstaens G., Camilleri M., Sifrim D., Houghton L.A., Elsenbruch S., Lindberg G., Azpiroz F., Parkman H.P. (2016). Fundamentals of Neurogastroenterology: Physiology/Motility—Sensation. Gastroenterology.

[9] Apidianakis Y., Rahme L.G. (2011). Drosophila Melanogaster as a Model for Human Intestinal Infection and Pathology. Dis. Model. Mech..

[10] Hadjieconomou D., King G., Gaspar P., Mineo A., Blackie L., Ameku T., Studd C., de Mendoza A., Diao F., White B.H. (2020). Enteric Neurons Increase Maternal Food Intake during Reproduction. Nature.

[11] Charalambous A., Grivogiannis E., Dieronitou I., Michael C., Rahme L., Apidianakis Y. (2022). Proteobacteria and Firmicutes Secreted Factors Exert Distinct Effects on *Pseudomonas aeruginosa* Infection under Normoxia or Mild Hypoxia. Metabolites.

[12] Pitsouli C., Apidianakis Y., Perrimon N. (2009). Homeostasis in Infected Epithelia: Stem Cells Take the Lead. Cell Host Microbe.

[13] LaJeunesse D.R., Johnson B., Presnell J.S., Catignas K.K., Zapotoczny G. (2010). Peristalsis in the Junction Region of the Drosophila Larval Midgut is Modulated by DH31 Expressing Enteroendocrine Cells. BMC Physiol..

[14] Benguettat O., Jneid R., Soltys J., Loudhaief R., Brun-Barale A., Osman D., Gallet A. (2018). The DH31/CGRP Enteroendocrine Peptide Triggers Intestinal Contractions Favoring the Elimination of Opportunistic Bacteria. PLoS Pathog..

[15] Chen J., Kim S.-M., Kwon J.Y. (2016). A Systematic Analysis of Drosophila Regulatory Peptide Expression in Enteroendocrine Cells. Mol. Cells.

[16] Coast G.M., Webster S.G., Schegg K.M., Tobe S.S., Schooley D.A. (2001). The Drosophila Melanogaster Homologue of an Insect Calcitonin-like Diuretic Peptide Stimulates V-ATPase Activity in Fruit Fly Malpighian Tubules. J. Exp. Biol..

[17] Wu Q., Zhao Z., Shen P. (2005). Regulation of Aversion to Noxious Food by Drosophila Neuropeptide Y- and Insulin-like Systems. Nat. Neurosci..

[18] Wu Q., Zhang Y., Xu J., Shen P. (2005). Regulation of Hunger-Driven Behaviors by Neural Ribosomal S6 Kinase in Drosophila. Proc. Natl. Acad. Sci. USA.

[19] Wu Q., Wen T., Lee G., Park J.H., Cai H.N., Shen P. (2003). Developmental Control of Foraging and Social Behavior by the Drosophila Neuropeptide Y-like System. Neuron.

[20] Yoshinari Y., Kosakamoto H., Kamiyama T., Hoshino R., Matsuoka R., Kondo S., Tanimoto H., Nakamura A., Obata F., Niwa R. (2021). The Sugar-Responsive Enteroendocrine Neuropeptide F Regulates Lipid Metabolism through Glucagon-like and Insulin-like Hormones in Drosophila Melanogaster. Nat. Commun..

[21] Chen J., Reiher W., Hermann-Luibl C., Sellami A., Cognigni P., Kondo S., Helfrich-Förster C., Veenstra J.A., Wegener C. (2016). Allatostatin A Signalling in Drosophila Regulates Feeding and Sleep and is Modulated by PDF. PLoS Genet..

[22] Hergarden A.C., Tayler T.D., Anderson D.J. (2012). Allatostatin-A Neurons Inhibit Feeding Behavior in Adult *Drosophila*. Proc. Natl. Acad. Sci. USA.

[23] Hentze J.L., Carlsson M.A., Kondo S., Nässel D.R., Rewitz K.F. (2015). The Neuropeptide Allatostatin A Regulates Metabolism and Feeding Decisions in Drosophila. Sci. Rep..

[24] Kubrak O., Koyama T., Ahrentløv N., Jensen L., Malita A., Naseem M.T., Lassen M., Nagy S., Texada M.J., Halberg K.V. (2022). The Gut Hormone Allatostatin C/Somatostatin Regulates Food Intake and Metabolic Homeostasis under Nutrient Stress. Nat. Commun..

[25] Chatterjee N., Perrimon N. (2021). What Fuels the Fly: Energy Metabolism in Drosophila and Its Application to the Study of Obesity and Diabetes. Sci. Adv..

[26] Shimizu Y., Matsuyama H., Shiina T., Takewaki T., Furness J.B. (2008). Tachykinins and Their Functions in the Gastrointestinal Tract. Cell. Mol. Life Sci..

[27] Apidianakis Y., Pitsouli C., Perrimon N., Rahme L. (2009). Synergy between Bacterial Infection and Genetic Predisposition in Intestinal Dysplasia. Proc. Natl. Acad. Sci. USA.

[28] Klindworth A., Pruesse E., Schweer T., Peplies J., Quast C., Horn M., Glöckner F.O. (2013). Evaluation of General 16S Ribosomal RNA Gene PCR Primers for Classical and Next-Generation Sequencing-Based Diversity Studies. Nucleic Acids Res..

[29] Mackay T.F.C., Richards S., Stone E.A., Barbadilla A., Ayroles J.F., Zhu D., Casillas S., Han Y., Magwire M.M., Cridland J.M. (2012). The Drosophila Melanogaster Genetic Reference Panel. Nature.

[30] Huang W., Massouras A., Inoue Y., Peiffer J., Ràmia M., Tarone A.M., Turlapati L., Zichner T., Zhu D., Lyman R.F. (2014). Natural Variation in Genome Architecture among 205 Drosophila Melanogaster Genetic Reference Panel Lines. Genome Res..

[31] Heys C., Lizé A., Blow F., White L., Darby A., Lewis Z.J. (2018). The Effect of Gut Microbiota Elimination in Drosophila Melanogaster: A How-to Guide for Host-Microbiota Studies. Ecol. Evol..

[32] Ridley E.V., Wong A.C.-N., Westmiller S., Douglas A.E. (2012). Impact of the Resident Microbiota on the Nutritional Phenotype of Drosophila Melanogaster. PLoS ONE.

[33] Clark A.G., Fucito C.D. (1998). Stress Tolerance and Metabolic Response to Stress in Drosophila Melanogaster. Heredity.

[34] Hill W.G., Goddard M.E., Visscher P.M. (2008). Data and Theory Point to Mainly Additive Genetic Variance for Complex Traits. PLoS Genet..

[35] Weng Y.-L., Liu N., DiAntonio A., Broihier H.T. (2011). The Cytoplasmic Adaptor Protein Caskin Mediates Lar Signal Transduction during Drosophila Motor Axon Guidance. J. Neurosci..

[36] Chung B.Y., Ro J., Hutter S.A., Miller K.M., Guduguntla L.S., Kondo S., Pletcher S.D. (2017). Drosophila Neuropeptide F Signaling Independently Regulates Feeding and Sleep-Wake Behavior. Cell Rep..

[37] Veenstra J.A., Agricola H.-J., Sellami A. (2008). Regulatory Peptides in Fruit Fly Midgut. Cell Tissue Res..

[38] Dutta D., Dobson A.J., Houtz P.L., Gläßer C., Revah J., Korzelius J., Patel P.H., Edgar B.A., Buchon N. (2015). Regional Cell-Specific Transcriptome Mapping Reveals Regulatory Complexity in the Adult Drosophila Midgut. Cell Rep..

[39] Broderick N.A., Lemaitre B. (2012). Gut-Associated Microbes of Drosophila Melanogaster. Gut Microbes.

[40] Chandler J.A., Morgan Lang J., Bhatnagar S., Eisen J.A., Kopp A. (2011). Bacterial Communities of Diverse Drosophila Species: Ecological Context of a Host–Microbe Model System. PLoS Genet..

[41] Rothschild D., Weissbrod O., Barkan E., Kurilshikov A., Korem T., Zeevi D., Costea P.I., Godneva A., Kalka I.N., Bar N. (2018). Environment Dominates over Host Genetics in Shaping Human Gut Microbiota. Nature.

[42] Cognigni P., Bailey A.P., Miguel-Aliaga I. (2011). Enteric Neurons and Systemic Signals Couple Nutritional and Reproductive Status with Intestinal Homeostasis. Cell Metab..

[43] Dus M., Lai J.S.-Y., Gunapala K.M., Min S., Tayler T.D., Hergarden A.C., Geraud E., Joseph C.M., Suh G.S.B. (2015). Nutrient Sensor in the Brain Directs the Action of the Brain-Gut Axis in Drosophila. Neuron.

[44] Lin S., Senapati B., Tsao C.-H. (2019). Neural Basis of Hunger-Driven Behaviour in Drosophila. Open Biol..

[45] Keene A.C., Duboué E.R., McDonald D.M., Dus M., Suh G.S.B., Waddell S., Blau J. (2010). Clock and Cycle Limit Starvation-Induced Sleep Loss in Drosophila. Curr. Biol..

[46] Chopra G., Kaushik S., Kain P. (2022). Nutrient Sensing via Gut in Drosophila Melanogaster. Int. J. Mol. Sci..

[47] Veenstra J.A., Ida T. (2014). More Drosophila Enteroendocrine Peptides: Orcokinin B and the CCHamides 1 and 2. Cell Tissue Res..

[48] Visscher P.M., Hill W.G., Wray N.R. (2008). Heritability in the Genomics Era—Concepts and Misconceptions. Nat. Rev. Genet..

[49] O’Connell K.S., Hindley G., Smeland O.B., Shadrin A., Wang Y., Frei O., Andreassen O.A., Tsermpini E.E., Alda M., Patrinos G.P.B.T.-P.G. (2022). Chapter 16—Shared Heritability among Psychiatric Disorders and Traits. Translational and Applied Genomics.

[50] Martin A.R., Daly M.J., Robinson E.B., Hyman S.E., Neale B.M. (2019). Predicting Polygenic Risk of Psychiatric Disorders. Biol. Psychiatry.

[51] Havdahl A., Niarchou M., Starnawska A., Uddin M., van der Merwe C., Warrier V. (2021). Genetic Contributions to Autism Spectrum Disorder. Psychol. Med..

[52] Blizard D.A., Adams N. (2002). The Maudsley Reactive and Nonreactive Strains: A New Perspective. Behav. Genet..

[53] Branicky R., Hekimi S. (2006). What Keeps C. Elegans Regular: The Genetics of Defecation. Trends Genet..

[54] Bonfiglio F., Liu X., Smillie C., Pandit A., Kurilshikov A., Bacigalupe R., Zheng T., Nim H., Garcia-Etxebarria K., Bujanda L. (2021). GWAS of Stool Frequency Provides Insights into Gastrointestinal Motility and Irritable Bowel Syndrome. Cell Genom..

[55] Lin H.-H., Kuang M.C., Hossain I., Xuan Y., Beebe L., Shepherd A.K., Rolandi M., Wang J.W. (2022). A Nutrient-Specific Gut Hormone Arbitrates between Courtship and Feeding. Nature.

[56] Jimenez-Sanchez G., Hebron K.J., Thomas G., Valle D. (1999). Targeted Disruption of the 70 kDa Peroxisomal Membrane Protein (PMP70) in Mouse is Associated with an Increase in the Related P70R Protein, Deficiency of Hepatic Glycogen and a Dicarboxylic Aciduria. Pediatr. Res..

[57] Imanaka T., Aihara K., Suzuki Y., Yokota S., Osumi T. (2000). The 70-KDa Peroxisomal Membrane Protein (PMP70) an ATP-Binding Cassette Transporter. Cell Biochem. Biophys..

[58] Schnorrer F., Schönbauer C., Langer C.C.H., Dietzl G., Novatchkova M., Schernhuber K., Fellner M., Azaryan A., Radolf M., Stark A. (2010). Systematic Genetic Analysis of Muscle Morphogenesis and Function in Drosophila. Nature.

[59] Hudson A.M., Petrella L.N., Tanaka A.J., Cooley L. (2008). Mononuclear Muscle Cells in Drosophila Ovaries Revealed by GFP Protein Traps. Dev. Biol..

[60] Vasmer D., Pooryasin A., Riemensperger T., Fiala A. (2014). Induction of Aversive Learning through Thermogenetic Activation of Kenyon Cell Ensembles in Drosophila. Front. Behav. Neurosci..

[61] Pang T.-L., Ding Z., Liang S.-B., Li L., Zhang B., Zhang Y., Fan Y.-J., Xu Y.-Z. (2021). Comprehensive Identification and Alternative Splicing of Microexons in Drosophila. Front. Genet..

[62] Tsao C.-H., Chen C.-C., Lin C.-H., Yang H.-Y., Lin S. (2018). Drosophila Mushroom Bodies Integrate Hunger and Satiety Signals to Control Innate Food-Seeking Behavior. Elife.

