# Supplementary material for: Dietary Stimuli, Intestinal Bacteria and Peptide Hormones Regulate Female Drosophila Defecation Rate
Source: Metabolites. 2023 Feb 12;13(2):264. doi: 10.3390/metabo13020264 (PMC9965912; doi:10.3390/metabo13020264)
Supplement: Supplementary file 1 [file metabolites-13-00264-s001.zip › Supplementary Figure S1.pdf]

Supplementary Figure S1.

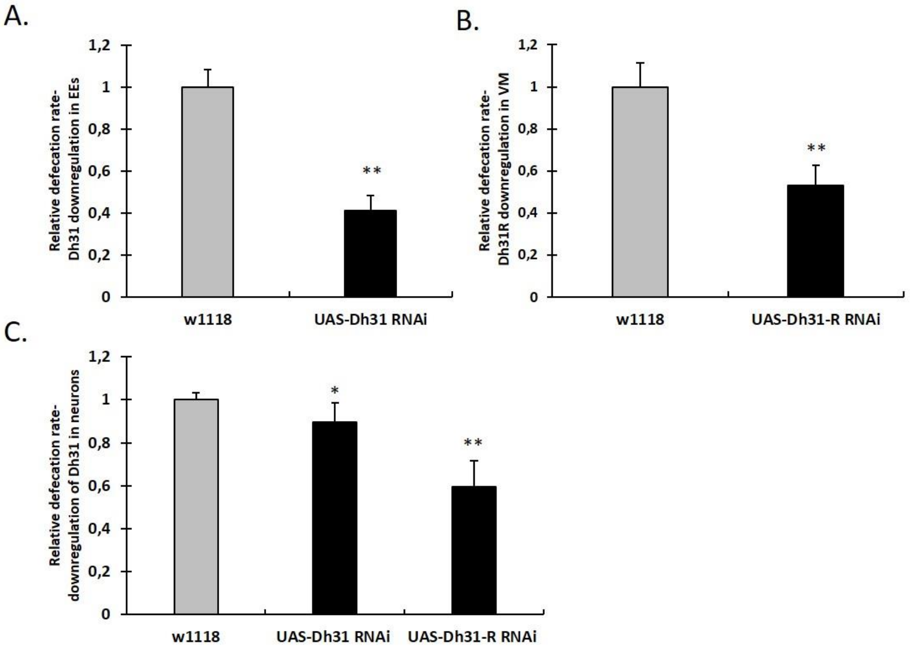

Supplementary Figure S1. Both downregulation of *Dh31* in EEs or neurons and downregulation of *Dh31*-receptor (Dh31-R) in Visceral Muscle (VM) or neurons reduces the defecation rate. Fecal spots per fly per day upon **(A)** *Dh31* downregulation in the midgut EEs, **(B)** *Dh31-R* downregulation in the VM (via 24B-Gal4-UAS-RNAi) and **(C)** *Dh31* and *Dh31*-R downregulation in all neurons, divided by that of the progeny of Gal4 crossed to w^{1118}. Each column represents the mean value of 6 biological replicates. Error bars represent standard deviation of the mean. Statistical significance using the Mann-Whitney U-test indicated as *p < 0.05 or **p<0.01.
