# Supplementary material for: Dietary Stimuli, Intestinal Bacteria and Peptide Hormones Regulate Female Drosophila Defecation Rate
Source: Metabolites. 2023 Feb 12;13(2):264. doi: 10.3390/metabo13020264 (PMC9965912; doi:10.3390/metabo13020264)
Supplement: Supplementary file 1 [file metabolites-13-00264-s001.zip › Supplementary Table S1.pdf]

Supplementary Table S1. Gene-specific primers for qPCR amplification. qPCR amplification was performed using gene-specific primers.

Genes	Primer Names	Primer Sequences
RPL32	Rpl32_F	CGGATCGATATGCTAAGCTGT
	Rpl32_R	CGACGCACTCTGTTGTCG
GAPDH	Gapdh1-RA_F	GCTCCGGGAAAAGGAAAA
	Gapdh1-RA_R	TCCGTTAATTCCGATCTTCG
AstA	AstA_F	CGCCTGCCGGTCTATAACTT
	AstA_R	CTTGTTCTGTCGGCCAGGTC
AstC	AstC_2F	CTCACCCTGTTCTTTGCCCT
	AstC_2R	GGTCCTGTTTCGGCACCC
Dh31	Dh31_2F	TGGCGCTGGCTATAACGAAC
	Dh31_2R	CCGTATGATGGTGCGTCCAA
NPF	NPF_2F	TCCGCGAAAGAACGATGTCA
	NPF_2R	CTGTCGCCGTAGTAGGTGTC
ckn	ckn_F	TCCACCGCACAGGCTAAGA
	ckn_R	GGCTGCTGATAGAGATGCAAATC
meso18E	meso18E_F	AGTGCTGCAATCGGTACGAG
	meso18E_R	GCTTGCCATTCGATCCCCA
Pmp70	Pmp70_F	TCCAACAAGAAGCGAAAACCC
	Pmp70_R	AGGAAGAGCAGTCCGGTCTC
CG11307	CG11307_F	CCGCGCTAAGTGAATGCTCT
	CG11307_R	AACGACCACTGTTCCAAAACT
mub	mub_2F	CCGGAACGTATTGTGACTGTG
	mub_2R	TCGAACTTCTTTGTAATGAGCGT
